# Understanding the impact of radiation-induced lymphopenia: Preclinical and clinical research perspectives

**DOI:** 10.1016/j.ctro.2024.100852

**Published:** 2024-09-06

**Authors:** E. Prades-Sagarra, A. Yaromina, L.J. Dubois

**Affiliations:** a The M-Lab, Department of Precision Medicine, GROW - Research Institute for Oncology and Reproduction, Maastricht University, 6229 ER Maastricht, The Netherlands

**Keywords:** Radiation-induced lymphopenia, Preclinical models, Immunotherapy

## Abstract

Immunotherapy has revolutionized the field of cancer treatment, changing the standard of care to the use of immune checkpoint inhibitors. Radiotherapy can boost anti-tumour immune responses by changing the tumour microenvironment, but it also can cause radiotherapy-induced lymphopenia (RIL), a decrease in circulating lymphocyte counts. RIL has been associated with lower survival in patients undergoing radiotherapy, and new studies have suggested that it can also affect immunotherapy outcome. To study RIL’s effects and to explore mitigation treatment strategies, preclinical models closely mimicking the clinical situation are needed. State-of-the-art image-guided small animal irradiators now offer the possibility to target specific organs in small animals to induce RIL, aiding research on its molecular mechanisms and prevention. This review covers the relationship between radiotherapy and RIL, its impact on patient survival, and future directions to generate models to investigate and prevent RIL.

## Highlights

• Radiotherapy can cause lymphopenia, a decrease in circulating lymphocytes.
• Radiation-induced lymphopenia is a negative prognostic factor in several solid tumours.
• Developing preclinical models is needed to understand lymphopenia’s impact on treatment outcome.
• Research should focus on lymphopenia prevention via pharmacological or technological strategies.
• Lymphopenia prevention could have a significant impact on the treatment of cancer patients.

## Immunotherapy and radiotherapy as a novel cancer combination treatment

Immunotherapy has reshaped the field of anti-cancer immune treatment, especially in the last decades. Immunotherapy was discovered in the 1890s when William Bradley Coley, now known as the father of immunotherapy, studied the relationship between the immune system and cancer. He found a correlation between bone sarcoma cancer remission and concomitant bacterial infection, which led him to inject different bacterial strains into the patients’ tumours to cure the disease and subsequently develop the first-known form of anti-cancer immunotherapy. However, the idea of injecting pathogenic bacteria to patients did not convince most of the medical community, and was subsequently forgotten for many decades [1]. It was until the late 1990s when this type of anti-cancer therapy regained the interest of the scientific community and changed the field of cancer treatment. It started with the FDA-approval of interleukin (IL) 2 against renal cell carcinoma in 1992, followed by the approval of the first monoclonal antibody to treat non-Hodgins lymphoma in 1997. This era continued with the discovery of several immunotherapeutic drugs, including the immune-checkpoint inhibitors (ICIs) ipilimumab, an antibody against Cytotoxic T-Lymphocyte-Associated Protein 4 (CTLA-4) and nivolumab, an antibody against Programmed cell Death protein 1 (PD-1), as well as ongoing clinical trials to exploit the relationship between the immune system and cancer [1], [2], [3].

Up to now, different immunotherapy strategies have been tested in preclinical models and/or clinical studies, which have shown beneficial effects in terms of patient prognosis and overall survival [4]. The blockage of CTLA-4 in cancer patients has been shown to lead to tumour regression in clinical trials including patients with melanoma, prostate and ovarian cancer [5]. Anti-PD-1 and anti-PD-L1 (Programmed Death Ligand 1) antibodies have also been suggested as immune checkpoint therapies preventing T cell exhaustion. Favourable clinical outcomes have been observed in patients treated with anti-PD-L1 and anti-PD-1 antibodies for non-small cell lung carcinoma (NSCLC) and melanoma, amongst others [5]. Interleukins, such as IL-2, IL-15 and IL-12, have been proposed as anti-cancer immunotherapeutic drugs due to their positive effect on T and NK cell expansion, maturation and function; however, its translation into the clinic has been hampered due to their side effects [6].

In the past decades, radiotherapy has become the standard treatment for more than half of cancer patients. Radiotherapy has direct anti-tumour effects via induction of DNA damage in cancer cells, which leads to mitotic catastrophe and cell death, but also indirect effects including damage in the tumour vasculature as well as priming an immune anti-tumour response [7]. Radiotherapy has been proposed to have an immunomodulatory effect by mimicking an “in situ vaccination” and triggering an anti-tumour immune response, which can be enhanced by the use of immunotherapy [8], [9], [10], [11]. For instance, radiation has been proposed to transform “cold tumours” with low immune cell infiltration into “hot tumours” by reprograming the tumour microenvironment and increasing immune cell infiltration, a key condition for the effectiveness of ICIs [8], [9], [11], [12]. In both preclinical models and clinical trials, local tumour irradiation enhanced the response to anti-CTLA-4, as well as to anti-PD-1 and/or anti-PD-L1 in preclinical studies [11], [12].

## The negative effects of radiotherapy: radiation-induced lymphopenia (RIL)

However, radiotherapy also has a negative impact on the immune system, as it causes direct adverse effects in the healthy tissue surrounding the tumour, including the kill of circulating immune cells in peripheral blood [8]. Radiotherapy is known to cause radiotherapy-induced lymphopenia (RIL), i.e. a decrease in lymphocyte counts [13], [14]. According to the Common Terminology Criteria for Adverse effects (CTCAE; version 5), RIL can be classified into four different grades depending on the severity: grade 1 < 1000–800 cells/µL; grade 2 500–800 cells/µL, with a minimum 20 % drop; grade 3 200–500 cells/µL, with a minimum 50 % drop; and grade 4 < 200 cells/µL, with a minimum 80 % drop [15].

Lymphocytes are known to be one of the most radiosensitive cells in the human body, being B cells the most sensitive subtype. *In vitro* studies showed that only 0.5 Gy caused a 10 % decrease in viability of human T lymphocytes, and 1.5 Gy induced more than 50 % cell death [16]. CD4^+^ T cells have been proposed to be more sensitive to irradiation than CD8^+^ T cells, followed by NK cells, which are the most radioresistant subtype [17]. Interestingly, T cell sensitivity to irradiation also varies depending on the location. *In vivo* studies have shown that CD8^+^ T cells located in lymphoid organs such as the spleen and lymph nodes were more sensitive to radiation, followed by liver T cells with intermediate sensitivity. Gut and intratumoural CD8^+^ T cells showed the most radioresistant resistant phenotype [18].

More than 40 % of patients with solid tumours receiving radiotherapy develop grade ≥ 3 RIL. Some risk factors for severe RIL have been described, including low baseline lymphocyte counts, aging, female sex and concomitant chemotherapy [19], [20], [21]. Radiation therapy associated factors could also play a role on the severity of RIL, including larger volumes, high number of fractions and especially higher doses to lymphocyte-rich organs such as heart, spleen and thoracic vertebrae as well as lung [20], [22], [23].

## Radiation-induced lymphopenia in the clinic

RIL has been extensively reported to be a negative prognostic factor in many solid tumour types [14], including glioblastoma [24], head and neck [25], lung [26], pancreas [27] and breast cancer [28]. The impact of RIL has also been linked with its severity. Grade ≥ 3 RIL has been associated with a 65 % increased risk of dead compared to grade 0–2 RIL in patients with solid tumours. In fact, when investigating the overall survival of patients with grade 4 RIL, these had a 50 % more risk of death compared to patients with grade 0–3 RIL [20].

As the therapeutic effect of immunotherapy drugs depends mainly on the status of lymphocytes, especially T cells, recent and current clinical studies are investigating the effect of RIL on the efficacy of immunotherapy in combination with radiotherapy (Table 1). Few retrospective studies showed that NSCLC patients experiencing RIL at time of start of immunotherapy had a worse progression free and overall survival compared with patients with normal absolute lymphocyte counts [22], [26], [29], [30], [31]. This was also observed in other retrospective trials including melanoma [22], [32], [33], [34], renal cell carcinoma [22], [35], and head and neck carcinoma patients [35], [36], [37], [38], showing that RIL at time of ICI administration was associated with lower survival. As these studies are retrospective, a causal relationship between RIL and worse outcome in patients treated with immunotherapy and radiotherapy cannot be assessed.

**Table 1:** Summary of published (retrospective) clinical studies evaluating the impact of lymphopenia on survival of patients treated with immune checkpoint inhibitors in combination with radiotherapy.

Cancer type	Treatment (incl. % patients)	Time of measure	Incidence & severity of lymphopenia	Impact on patient survival	Ref.
Solid tumours (mainly NSCLC, melanoma)	- Prior RT or chemotherapy - ICI (mainly pembrolizumab 50 %, nivolumab 40 %, atezolizumab 5 %, nivolumab + ipilimumab 4 %)	ALC at 3 months post-start of ICI	- Grade 1–2 31 %, Grade ≥ 3 5 %	- Grade ≥ 3: shorter PFS (5.3 vs 15.1 months) & OS (9.8 vs 18.3 months)	[21]
Solid tumours (lung, renal cell and melanoma)	- RT (Prior and/or adjuvant; 100 %) - Prior chemotherapy (≈20 %) - Pembrolizumab or nivolumab (100 %) - Ipilimumab (concurrent 14 %; adjuvant 21 %)	ALC at start of anti-PD-1	- Grade ≥ 3 ≈17 %	- Grade ≥ 3: lower median survival (157 vs 363 days)	[22]
Solid tumours (mainly lung, melanoma, renal, head and neck)	- Prior RT (51 %) or chemotherapy (75 %) - Nivolumab (75 %) or pembrolizumab (25 %) - Ipilimumab (prior 11 %, concurrent 17 %)	ALC at 3 months post-start of ICI	- Grade ≥ 1 ≈30 %	- Grade ≥ 1: worse PFS	[35]
Solid tumours (mainly lung, head and neck, renal)	- RT (100 %; mostly concurrent; + prior ≈28 %) - Prior chemotherapy (≈93 %) - ICI (ipilimumab 64 %, pembrolizumab 36 %; concurrent + adjuvant post-RT)	ALC post-RT, and start of ICI	- Median ALC=560cells/µL (Grade 2)	- Grade ≥ 2 (<560cells/µL): worse PFS (6.0 vs 12.3 months) and OS (15.7 vs 27.4 months)	[37]
Lung (NSCLC)	- RT (prior 44 %, concurrent 25 %) - Prior chemotherapy (≈96 %) - Nivolumab, pembrolizumab or nivolumab + ipilimumab	Peri-immunotherapy ALC (2 weeks before start to 6 months from start of ICI)	- Grade ≥ 1 ≈55 %	- Grade ≥ 1: worse PFS (2.2 vs 5.9 months) and OS (5.7 vs 12.1 months)	[26]
Lung (NSCLC)	- RT with concurrent chemotherapy (100 %) - Durvalumab, pembrolizumab or nivolumab (≈38 %; as consolidation treatment)	ALC at baseline and overtime post-start of RT	- Grade ≥ 3 ≈77 %	- Group with high incidence Grade ≥ 3: worse PFS and OS than lower incidence of grade ≥ 3 groups - Only subgroup consolidative immunotherapy: lower PFS and OS in high incidence grade ≥ 3 compared to low incidence of grade ≥ 3 groups - In low incidence of grade ≥ 3 RIL groups, consolidative immunotherapy improved PFS and OR; in high incidence of grade 3, ICI was not significantly associated with PFS and OR	[29]
Lung (NSCLC)	- RT with concurrent chemotherapy - Durvalumab (94 %) or ipilimumab + nivolumab (5 %); adjuvant	ALC at start of ICI	- Grade ≥ 3 23 %	- Grade ≥ 3: worse PFS (217 vs 570 days)	[30]
Lung (NSCLC)	- Chemoradiotherapy (100 %), of which 75 % received proton RT - Durvalumab (38 %)	ALC over time during treatment	- Grade ≥ 3 32 %	- Grade ≥ 3: worse PFS (17.1 vs 19.7 months) and OS (24.6 months vs not reached)	[31]
Lung (NSCLC)	- Chemoradiotherapy (100 %) - Durvalumab (94 %) or pembrolizumab (6 %); adjuvant	ALC after chemoradiation	- Grade 3 ≈49 %, grade 4 ≈11 % - Persistent grade ≥ 3 at 3 months after chemoradiotherapy (≈11 %)	- Persistent grade ≥ 3: lower PFS (6.1 vs 23.1 months) and OS (13.9 months vs not reached), compared to recovered lymphopenia after 3 months of chemoradiation	[39]
Melanoma	- Prior RT (51 %) or chemotherapy (75 %) - Ipilimumab, nivolumab, pembrolizumab, and/or ipilimumab + nivolumab	ALC at baseline and over time until 6 months post-start of ICI*	- Grade ≥ 1 ≈42 % - Mean onset 17 weeks post-start ICI	- Grade ≥ 1: lower PFS (13.3 vs 16.9 months) and OS (28.1 vs 36.8 months) - Early onset (<4 months post-start ICI) = lower OS than late onset (>4 months post-start ICI)	[32]
Melanoma	- Prior RT (≈18 %), chemotherapy (≈31 %) or other immunotherapies (≈20 %) - Nivolumab (100 %)	ALC after 1st and 2nd dose	- Grade ≥ 1	- Lower OS when grade ≥ 1 at 1st and 2nd dose	[33]
Melanoma	- Prior RT (40 %), chemotherapy (93 %) or others - Ipilimumab (100 %)	ALC after 1st and 2nd dose; OS at week 24 post-start of ICI	- Grade ≥ 1 (≈20 % and ≈16 % after 1st and 2nd dose)	- After 1st dose: Grade ≥ 1 = worse OS (1.8 vs 7.9 months) - After 2nd dose: Grade ≥ 1 = worse OS (1.4 vs 11.9 months)	[34]
Head and neck squamous cell carcinoma	- Surgery (60 %), chemotherapy (67 %), radiotherapy (89 %) or chemoradiotherapy (concurrent, 65 %) - Nivolumab (100 %)	ALC at start of ICI and over time	- Grade ≥ 1 56 %, of which Grade ≥ 3 13 % at start of ICI - Persistent Grade ≥ 1 during ICI treatment 41 %	- Persistent grade 1 lymphopenia during ICI treatment: worse OS (5.5 vs 15.6 in patients with resolving lymphopenia vs 11.2 months in patients with no lymphopenia), - No effect on PFS	[36]
Head and neck squamous cell carcinoma	- Prior chemoradiotherapy (≥2 lines, ≈47 %) - Nivolumab (≈47 %), pembrolizumab (≈53 %)	ALC at start of ICI	- Grade ≥ 2 ≈38 %	- Grade ≥ 2: worse PFS (60 vs 141 days)	[38]

ALC − absolute lymphocyte counts; OS − overall survival; PFS − progression free survival, RT − radiotherapy. Ipilimumab (CTLA-4 inhibitor); pembrolizumab, nivolumab (PD-1 inhibitors); atezolizumab, durvalumab (PD-L1 inhibitors). *Only patients with normal baseline ALC were included.

Clinical studies showed that chemotherapy-induced lymphopenia has a shorter duration, while RIL can last for several months up to years to resolve [13]. This could be explained due to the inability to activate the usual compensatory mechanism based on the rise of IL-7 levels to induce lymphocyte expansion and both IL-7 and IL-15 to induce lymphocyte maturation after radiotherapy, contributing to the chronicity of RIL [14].

## The lack of preclinical RIL models

Surprisingly, preclinical studies investigating the effects of lymphopenia on treatment outcome are sparse, possibly explained by the current lack of RIL preclinical models. In the past decades, the preclinical models used to study the immune system and RIL were generated by knocking out essential genes for T cell expansion, maturation and/or activity, such as the IL-2 receptor, or by total body irradiation [40]. However, these models do not mimic the clinic situation in which patients develop RIL due to local irradiation treatment.

Few attempts have been carried out to establish RIL in mice using a clinically relevant setting, such as irradiation of the tumour [41] or normal tissues including heart, spleen [42], lymph nodes [43], [44] or brain [45] (Table 2). Fractionated tumour irradiation in a subcutaneous mouse model of hepatocellular carcinoma led to a 20 % drop in circulating CD3^+^ cells up to 7 days post-irradiation, suggesting a grade 2 RIL [41]. Similarly, irradiation of subcutaneous implanted colorectal tumour simultaneously with draining lymph nodes (DLN) and an additional abdominal region resulted in more than 50 % decrease in circulating lymphocytes 1 day post-irradiation (grade 3 RIL) and more than 20 % decrease up to 9 days post-irradiation [43]. A similar study was performed investigating the effects of irradiation of tumour with or without DLN irradiation in combination with immunotherapy (CTLA-4 inhibitor) in a melanoma mouse model [44]. DLN irradiation, either concomitant or neo-adjuvant (albeit prior) to ICI treatment led to decreased lymph node CD45^+^, CD8^+^, CD4^+^ and regulatory T cell counts. Interestingly, DLN receiving irradiation showed an increase in proliferating CD8^+^ cells compared to non-irradiated DLN, in line with the previously described compensatory lymphopenia-induced proliferation [44].

**Table 2:** Published preclinical murine RIL models and their impact on treatment outcome.

Strain	Target IR	Treatment	Incidence & severity of lymphopenia	Cancer	Relevance or impact	Ref.
BALB/c	Heart	- 5x2Gy or 1x8Gy - Ultra-high (35 Gy/sec) vs conventional dose rate	In both single dose or fractionated: - Grade ≥ 3 RIL: >50 % decrease in circulating CD3^+^, CD4^+^, CD8^+^, CD19^+^ counts up to day 17 post-IR in FLASH and conventional - ↑ Severity RIL with FLASH RT: grade 4 vs grade 3 RIL. - RIL sustained over time with FLASH: at day 24 post-IR, 50 % CD3^+^ with FLASH vs 100 % recovery with conventional	No	−	[42]
BALB/c	Spleen	- 5x1Gy or 1x5Gy - Ultra-high (35 Gy/sec) vs conventional dose rate	- In fractionated setting → Grade ≥ 3 RIL: >50 % decrease in CD3^+^ cells. Similar decrease in circulating CD4^+^, CD8^+^, CD19^+^ counts up to day 17 post-IR in FLASH and conventional at 3 days post-IR - FLASH: lower CD3^+^, CD4^+^, CD8^+^, CD19^+^ counts than conventional when single dose at 3 days post-IR	No	−	[42]
C57BL/6J	Brain	- Focal IR - 10x4Gy or 30x2Gy	- 10x4Gy → Grade ≥ 3 RIL (>50 % decrease in CD3^+^) one week post-IR. Similar decrease in CD45^+^, CD4^+^, CD8^+^ & Treg cells - 30x2Gy → Grade ≥ 3 RIL (>50 % decrease in CD3^+^) six week post-IR. Similar decrease in CD45^+^, CD4^+^, CD8^+^ & Treg cells	No	Altered architecture inguinal LN 10x4Gy: ↑ serum IL-7 (inverse correlation with total lymphocyte counts)	[45]
BALB/c	Brain	- Focal IR - 10x4Gy or 30x2Gy	- 10x4Gy → Grade ≥ 3 RIL (>30 % decrease in CD3^+^) one week post-IR. Similar decrease in CD45^+^, CD4^+^, CD8^+^ & Treg cells - 30x2Gy → Grade ≥ 3 RIL (≈50 % decrease in CD3^+^) one week post-IR. Similar decrease in CD45^+^, CD4^+^, CD8^+^ & Treg cells	No	Altered architecture inguinal LN*	[45]
C3H/HeN	Right hind limbs or tumour	- 1x15Gy or 10x3Gy - IL-7 (10 mg/kg; s.c.), at day of last IR fraction	In naïve animals: - Fractionated IR→lower PBMC counts compared to single dose In naïve and tumour-bearing animals: - Fractionated IR→Grade ≈2 RIL two days post-IR. Decrease of 20 % in CD45^+^; similar decrease in CD3^+^, CD8^+^, CD4^+^ & CD19^+^ cells - IL-7 administration → faster restoring & higher levels of CD45^+^, CD3^+^, CD8^+^, CD4^+^ & CD19^+^ cells - ↑ infiltration of CD4^+^ & CD8^+^ cells in tumour in IL-7-treated animals	Murine HCC (HCa-1; s.c.)	IL-7 levels in mesenteric LN→inversely correlated with CD45 cells in all groups IL-7 + IR→reduction in tumour growth compared to IR alone	[41]
C57BL/6J	Tumour and/or DLN and/or abdomen	- Tumour (12 Gy) - DLN (x2, 6 Gy/DLN) - Abdomen (x2, 6 Gy/region)	- Small IR volumes (tumour; tumour + DLN): decrease in circulating lymphocytes at day 1, and recovery at day 7 post-IR - Large IR volumes (tumour + abdomen; tumour + DLN+abdomen): stronger decrease in circulating lymphocyte counts (Grade ≈3 RIL: >50 % decrease in lymphocytes 1 day post-IR), more prolonged in time.	Murine colorectal (MC38; s.c.)	RIL doesn’t affect tumour response to radiotherapy (PFS, OS)	[43]
C57BL/6J	Tumour ± DLN	- Tumour (15 Gy) - DLN (x2, 15 Gy/DLN) - ICI (anti-CTLA-4; 200 μg/mouse, i.p.)	Lymphocyte counts in the DLN (not circulating): - DLN IR (concomitant or before ICI): >20 % decrease in CD45^+^, CD8^+^, CD4^+^ & Treg cells	Murine melanoma (B16F10-Fluc-Puro; s.c.)	DLN IR before ICI or concomitant: abrogates the effect of ICI+IR in tumour growth delay DLN IR post-ICI: preserved the effect of ICI+IR in tumour growth delay	[44]

IR − irradiation; LN − lymph node; DLN − draining lymph node; s.c. − subcutaneous; TBI − total body irradiation; CD45^+^ − leukocytes; CD3^+^ − T cells; CD4^+^ − helper T cells; CD8^+^ − cytotoxic T cells; CD19^+^ − B cells; Treg − regulatory T cells; PBMC − peripheral blood mononuclear cells; HCC − hepatocellular carcinoma; ICI − immune checkpoint inhibitor; i.p. − intraperitoneally. *IL-7 levels not tested.

In case of heart irradiation, both FLASH and conventional irradiation (in single or fractionated setting) led to grade 3 RIL with a more than 50 % drop in circulating CD3^+^ cells up to 17 days post-irradiation, and grade 2 RIL at 24 days post-irradiation [42]. Similarly, spleen irradiation using a comparable irradiation setting led to grade 3 RIL at day 3 post-irradiation [42]. Focal brain irradiation, using a single dose or fractionated setting, resulted in more than 30 % and 50 % drop in circulating CD3^+^ cells, respectively [45].

However, the impact of RIL on tumour response to radiotherapy and/or in combination with immunotherapy has, so far, not been extensively investigated in these models. To our knowledge, only one published study investigated this matter and showed that concomitant or neoadjuvant DLN irradiation, which led to decreased lymphocyte counts in the lymph nodes, abrogated the effect of ICI combined with tumour radiation [44]. Taking into account this evidence, one can conclude that there is a lack of studies investigating the causal relationship between RIL and treatment outcome. Therefore, research is warranted to establish RIL models using clinically relevant approaches to unravel this relationship and to investigate the effectiveness of RIL mitigation and prevention treatments.

## Preclinical RIL models

In order to develop preclinical RIL models that mimic the clinic situation, we consider that certain guidelines based on clinical evidence should be taken into account. Clinical trials have shown that irradiation of great pools of blood containing high counts of circulating lymphocytes, such as large blood vessels, spleen or heart, as well as hematopoietic sites (i.e. bones) are correlated with severity of RIL [46], [47]. On the contrary, irradiation of other organs such as liver and kidney in metastatic patients did not induce a decrease in lymphocyte counts [46]. Similarly to the spleen, lymph nodes contain large pools of lymphocytes and represent the typical sites of immune response generation via T cell priming and high tumour antigen load [48].

**Fig. 1:**
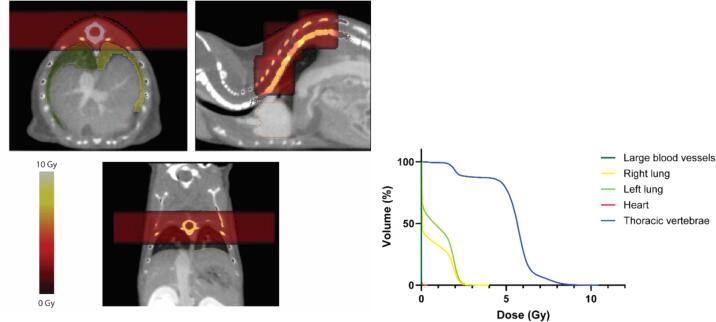
**Example of an irradiation plan targeting thoracic vertebrae to develop murine RIL models.** (Left) Representative example of mouse chest CT image with a treatment plan designed with the software smART-ATP (SmART Scientific Solutions B.V.). Three 5 mm square beams were positioned to target the thoracic vertebrae, each consisting of two 1 Gy parallel opposed lateral beams. Dose deposition is shown by colour scale. (Right) Resulting cumulative dose-volume histogram for treatment plan depicted in left panel, indicating a maximal coverage of the target region, while a minimal dose to surrounding tissues.

Taken this information into account, novel preclinical RIL models should be developed by irradiating heart, large blood vessels, spleen, bones or lymph nodes, ideally in a fractionated setting. By doing this, the models will mimic and reflect very closely the clinic situation [13]. Irradiation of such small structures in rodents to achieve reliable RIL is currently gaining attention due to the technological developments of high-precision small animal irradiators [49]. Based on CT imaging, these devices allow the design of a treatment plan adapted per animal, precise enough to mimic partial or total targeted organ irradiation. In case of vertebrae, simulations have been carried to investigate the feasibility to irradiate a part of the spinal cord, and subsequently the vertebrae, in mice, and showed that a dose painting approach offered the best coverage of the target volume with the lowest dose deposition in the surrounding tissues. However, the time required for the dose calculations and beam-on time was approximately 1 h, which is too long for an *in vivo* experiment in the absence of very accurate repositioning routine [50]. We have designed a new irradiation plan, which includes three 5 mm square beams, each with two parallel opposed radiation directions, showing a good coverage of the target organ and low dose deposition in the surrounding tissues (Fig. 1; Video 1). This novel plan requires a total calculation and beam-on time of less than 20 min, demonstrating the feasibility of the irradiation of such small structures in animal models.

## Pharmacological strategies to revert and/or prevent RIL

Pharmacological strategies, including interleukin administration, have been proposed to prevent RIL. Such strategies have already been assessed in patients with other immune diseases such as HIV-infected “non-responders”, virus-induced chronic hepatitis and patients receiving hematopoietic stem cell transplantation. Some of the most promising interleukins tested are IL-2, IL-15 and IL-7 owing to their role in T cell homeostasis [51], [52]. IL-2 is crucial for T and NK cell expansion and maturation as well as regulatory T cells function, and has also been proposed as an immunotherapeutic drug against certain cancers [53]. In line with IL-2, IL-15 stimulates the proliferation of T and NK cells and could be used as anti-tumour drug with successful anti-tumour effects [54], [55]. IL-7 plays a role in T cell homeostasis via regulating the development and maintenance of both T and B cells, and its use as anti-tumour immunotherapy has been tested in preclinical and clinical trials, however with inconclusive results [56], [57]. Previous studies on preclinical RIL models induced by focal brain irradiation showed an increase of IL-7 in serum, which was inversely correlated to the total lymphocyte counts, and could be speculated to be a compensatory mechanism to restore lymphocyte levels [45].

**Fig. 2:**
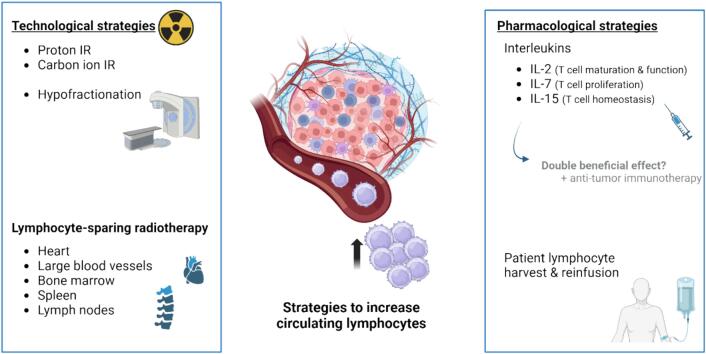
Strategies currently under investigation to prevent, reverse or mitigate RIL. Created with BioRender.com.

Only few studies have investigated the effects of these cytokines on RIL rescue (Fig. 2). Administration of IL-7 in mouse glioma models was observed to mitigate RIL via increasing both systemic and tumour CD8^+^ T cells, which presumably led to increased mouse survival [58]. Exogenous IL-7 administration has also been shown to recover circulating lymphocyte counts as well as T cell tumour infiltration in a mouse model of hepatocellular carcinoma after tumour-bearing hind leg irradiation. Furthermore, IL-7 administration led to a delayed tumour growth and increased survival compared to control [41]. A phase II clinical trial was carried investigating whether the administration of recombinant IL-7 was able to restore lymphocyte counts in breast cancer patients with lymphopenia. It is important to note that, in this case, patients did not receive radiotherapy, but chemotherapy. IL-7 increased both CD4^+^ and CD8^+^ cell counts when administered concomitantly with chemotherapy, and more prominently when administered prior to chemotherapy [59]. However, no differences were observed in patient survival between treatment groups [59]. Another phase I clinical trial also studied the use of IL-7 in glioma patients after chemoradiotherapy, and preliminary results showed a dose-dependent increase in absolute lymphocyte counts upon IL-7 administration [60]. Notably, small sample size was a limitation for both studies, and further confirmation of such results is warranted in larger clinical trials.

Considering these promising preliminary results, further research is needed to develop drugs which prevent or restore RIL and, in turn, improve tumour control and treatment outcome. One could also speculate that, if IL-2 and/or IL-15 exogenous administration is able to recover the absolute lymphocyte counts after radiation therapy, these treatments could have a double beneficial effect in cancer patients. On one hand, these interleukins could restore or even prevent the radiation-induced decrease in lymphocyte counts, while, on the other hand, enhance the anti-tumour immune response as immunotherapeutic drugs, considering they have been shown to have a beneficial effect in terms of tumour control in several types of cancer *in vivo* and in clinical trials. For example, the use of IL-2, combined with the carrier L19 (L19-IL2) to enhance tumour specific penetration, has been shown to improve survival in mouse models [9], [10], [11] and is currently being studied in a phase II clinical trial [61].

Another suggested strategy to restore lymphocyte counts after treatment is the harvest and reinfusion of patient lymphocytes. A clinical study investigated the possibility of harvesting lymphocytes prior to chemoradiotherapy in patients with high grade glioma. Next, patients were treated with chemoradiotherapy, which led to grade ≥ 3 RIL. After treatment, lymphocytes were reinfused, leading to a significant increase in both CD4^+^ and CD8^+^ T lymphocytes [62].

## Redefining radiation delivery techniques to protect lymphocytes and prevent RIL

Technological strategies suggested to decrease RIL include the use of different radiation types (Fig. 2), such as proton radiotherapy, which has been proposed to spare the circulating lymphocytes better compared to conventional photon radiotherapy [63], [64], [65], [66]. The effects of carbon ion radiation in RIL have also been described in retrospective studies. Patients receiving photon irradiation showed lower absolute lymphocyte counts and higher RIL severity compared to patients receiving carbon ion radiation treatment. These differences were also associated with changes in patient survival, with an increased survival of patients treated with carbon ion radiation [67]. To our knowledge, only one study investigated the “FLASH effect” on preclinical RIL models, and could not observe any protective effect of FLASH irradiation in terms of lymphocyte counts compared to conventional irradiation [42]. Additionally, the impact of fractionated radiation in circulating lymphocyte counts has been studied by means of a mathematical models based on data of patients with hepatocellular carcinoma. Shorter regimens and short interval times between fractions as well as between different regimens were predicted to increase and accelerate the recovery of circulating lymphocyte counts [68].

In addition, the establishment of clear guidelines for radiotherapy planning concerning the dose to the lymphocyte-related organs could help to prevent RIL and in turn improve clinical outcome (Fig. 2). Previous studies have discussed the feasibility of introducing constraints to protect these organs, a new concept named “lymphocyte sparing radiotherapy”, without compromising the tumour volume dose [13], [69].

## Conclusions

**Fig. 3:**
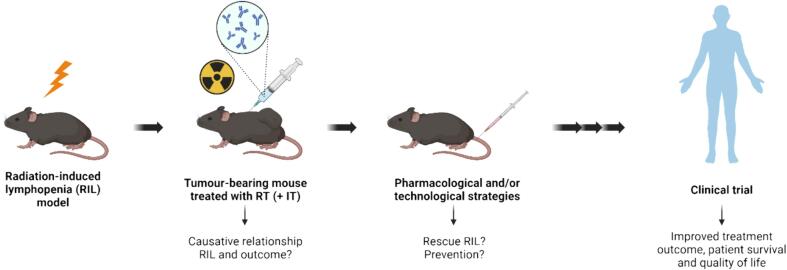
Roadmap for the study of the effects of RIL on tumour progression and strategies to prevent or reverse it to improve patient survival. RT − radiotherapy; IT − immunotherapy. Created with BioRender.com.

In conclusion, RIL is a negative prognostic factor in many solid tumour types. RIL may affect the efficacy of immunotherapy, observed by few studies showing decreased patient survival in patients with RIL when receiving radiotherapy and immunotherapy. Therefore, further research is warranted to study this interaction. The development of radiation-induced RIL preclinical models could help to establish a causative relationship between RIL and immunotherapy combined with radiotherapy, to investigate potential strategies to rescue RIL and have therefore a significant impact on the current standard of care for cancer patients (Fig. 3).

## Funding

E.P.S. has received funding from the European’s Union Horizon 2020 research and innovation program under the Marie Skłodowska-Curie grant agreement N°#860245 (granted to L.J.D.).

## Declaration of Competing Interest

The authors declare the following financial interests/personal relationships which may be considered as potential competing interests: L.J.D. has, outside of the submitted work, shares in the companies Convert Pharmaceuticals and LivingMed Biotech, he is co-inventor of a non-issued patent on LSRT (N2024889). The other authors declare no conflict of interest.
